# Coronary artery occlusions diagnosed by transthoracic Doppler

**DOI:** 10.1186/1476-7120-12-12

**Published:** 2014-03-15

**Authors:** Johnny Vegsundvåg, Espen Holte, Rune Wiseth, Knut Hegbom, Torstein Hole

**Affiliations:** 1 Department of Internal Medicine, Ålesund Hospital, Ålesund, Norway; 2 Department of Circulation and Medical Imaging, Norwegian University of Science and Technology (NTNU), Trondheim, Norway; 3 Department of Cardiology, Trondheim University Hospital, Trondheim, Norway; 4 Medical Faculty, Norwegian University of Science and Technology (NTNU), Trondheim, Norway; 5 Cardiology Division, Medical Department, Ålesund Hospital, 6026 Ålesund, Norway

**Keywords:** Transthoracic echocardiography, Coronary occlusion, Collateral flow

## Abstract

**Background:**

Our aim was to assess whether anterograde flow velocities in septal perforating branches could identify an occluded contralateral coronary artery, and to assess the feasibility and accuracy of diagnosing occlusions in the three main coronary arteries by the combined use of several noninvasive parameters indicating collateral flow.

**Methods:**

A total of 108 patients scheduled for coronary angiography because of chest pain or acute coronary syndromes were studied using transthoracic Doppler echocardiography.

**Results:**

Anterograde peak diastolic flow velocities (pDV) in septal perforating branches were higher in patients with angiographic occluded contralateral artery compared with corresponding velocities in patients without significant disease in the contralateral artery (0.80 ± 0.31 m/sec versus 0.37 ± 0.13 m/sec, p < 0.001). Receiver operating characteristic curve showed pDV ≥ 0.57 m/sec to be the optimal cutoff value to identify occluded contralateral artery, with a sensitivity of 79% and a specificity of 69%. Demonstration of at least one positive parameter (retrograde flow in main coronary arteries, reversed flow in septal perforating and left circumflex marginal branches, pDV ≥ 0.57 m/sec, or demonstration of other epicardial or intramyocardial collaterals) indicating collateral flow to an occluded main coronary artery had sensitivity, specificity, positive and negative predictive value of 89%, 94%, 63%, and 99%, respectively, for detection of a coronary occlusion. With this combined use of several parameters, 25 of 28 coronary occlusions were identified.

**Conclusions:**

By investigating several parameters indicating collateral flow, we were able to identify most of the main coronary occlusions in the patient cohort. Furthermore, our study demonstrated that coronary artery occlusions may result in complex and diverging coronary pathophysiology depending on which coronary artery segment is occluded and the extent of accompanying coronary artery disease.

**Trial registration:**

ClinicalTrials.gov number NTC00281346.

## Background

Coronary artery occlusions are common in coronary disease, with up to a quarter to a third of patients referred for coronary angiography reported to have coronary occlusions [1,2]. Occluded coronary arteries are associated with high incidence of cardiac events [3-5]. The coronary arteries are interconnected by intramyocardial and epicardial collaterals [6,7]. These preformed, small vessels have the potential to remodel and grow in response to ischemia, delivering blood by an alternative route to an ischemic territory [6,7]. Coronary lesion severity, proximal lesion location, and duration of ischemia are major determinants for collateralization [8,9]. Though collateral development starts early after the manifestation of ischemia, adequate collateralization may take several weeks [9]. There is individual variability in the propensity to develop collaterals. Coronary collaterals may significantly mitigate the effects of severe stenoses or occlusions [10-12]. A recent meta-analysis demonstrated a 36% mortality reduction in patients with high collateralization compared with patients with low collateralization [6].

Traditionally, coronary occlusions and collaterals have been assessed using selective coronary angiography [1,2,6,12]. Though transthoracic Doppler echocardiography (TTE) cannot give a complete and panoramic view of the coronary arteries and collateral vessels, various findings by TTE are shown to diagnose occlusions and collaterals with a high degree of accuracy [10,13-18]. Using TTE, a coronary occlusion may be detected by demonstrating retrograde flow in the arterial trunk, left circumflex marginal branches (CxMb), or septal perforating branches [10,13-17]. Enhanced flow in elongated epicardial or intramyocardial vessels has been shown to represent collateral flow to an occluded coronary artery [10,18]. Finally, accelerated anterograde flow velocities in septal perforating branches have been proposed to indicate collateral flow to an occluded artery [17,18]. However, these TTE studies are few, and most studies have in limited patient cohorts used only one or two parameters to detect coronary occlusions and collateral flow. Furthermore, anterograde flow velocities in septal perforators with collateral supply to occluded coronary arteries have not been extensively evaluated.

The purpose of our study was twofold. First, to assess whether anterograde flow velocities in septal perforating branches could identify an occluded contralateral coronary artery. Second, to assess the feasibility and accuracy of demonstrating occlusions in the three main coronary arteries by the combined use of several parameters, each of which indicates collateral flow. Coronary angiography was used as the reference for coronary occlusions.

## Methods

### Study population

Patients were included in the study if they fulfilled the following criteria: (1) already scheduled for coronary angiography because of documented or suspected stable or unstable coronary disease; (2) age above 18 years; (3) met no exclusion criteria. The exclusion criteria were: (1) previous aorto-coronary bypass surgery; (2) presumed insufficient acoustic windows because of severe emphysema or severe overweight; (3) significant valvular disease; (4) atrial fibrillation; (5) administrative reasons.

The study protocol was approved by the Regional Committee for Medical and Health Research Ethics and the Norwegian Data Inspectorate. All participants gave written, informed consent. (This study is registered at ClinicalTrials.gov under identifier NTC00281346).

Patient recruitment was prospective during the period 2006 – 2007 and all patients were from the local area of Ålesund Hospital. Six patients did not enter the study population because of insufficient acoustic windows (n = 3), lack of consent (n = 2) or aortic stenosis (n = 1). We included 115 patients in the study cohort, but seven patients were later excluded from further analysis because of protocol violation: aortic stenosis (n = 2), atrial fibrillation (n = 2), patient refusal of coronary angiography (n = 2), and no indication for coronary angiography (n = 1). The final cohort entering the study consisted of 108 patients. Baseline characteristics of the patients are presented in Table 1. All patients took their medication on the day of the echocardiographic study.

**Table 1:** Baseline characteristics of the study cohort (n = 108)

	**No of subjects (%) mean ± SD**
Age (years)	63.1 ± 9.5
Heart rate (beats/minute)	63 ± 7.4
BMI (kg/m^2^)	26 ± 3.6
Male sex	79 (73)
Total cholesterol (mmol/L)	4.9 ± 1.1
Blood pressure (mm Hg)	
Systolic	142 ± 20
Diastolic	82 ± 12
Medical history	
Hypertension (>140/90 mm Hg)	61 (55)
Current smoking	29 (27)
Diabetes	11 (10)
Previous CAD	23 (21)
ACS	35 (32)
Cardiac medication	
Aspirin	97 (90)
Thienopyridine	38 (35)
Low-molecular-weight heparin	30 (28)
β-Blockers	85 (79)
Statins	87 (81)
Calcium antagonists	21 (19)
ACE-inhibitors/ARB	24 (22)
Organic nitrate, daily maintenance	13 (12)

*BMI* body mass index, *CAD* coronary artery disease, *ACS* acute coronary syndromes, *ACE* angiotensin-converting enzyme, *ARB* angiotensin II receptor blocker.

### Transthoracic coronary flow evaluation

Patients were examined using an Acuson Sequoia C512 (Siemens Medical Solutions USA, Inc, Mountain View, CA) ultrasound system connected to standard 4V1C and 7V3C transthoracic transducers. Contrast agent was not used. The TTE examination was not performed earlier than the day after hospital admission and only after the patients were clinically stable. The coronary arteries were investigated by use of colour Doppler mapping with data postprocessing mix function, which makes the colours transparent, as described previously [19]. The Nyquist limit of colour Doppler was set to 0.24 m/sec, but was actively changed to provide optimal images. The colour box size was reduced to maintain the high frame rate. Stop-motion frames and clips were digitally recorded for offline analysis.

**Figure 1:**
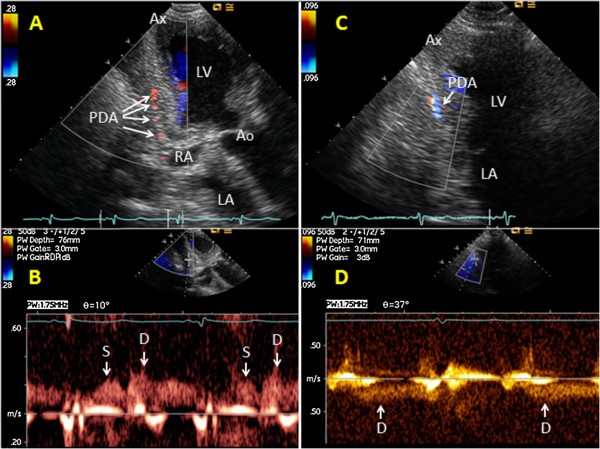
**Examples of anterograde and retrograde flow in the posterior descending coronary artery.** In modified apical long-axis views focusing on the posterior interventricular sulcus, the posterior descending coronary artery (PDA) is imaged by colour Doppler mapping with matching spectral Doppler tracings of blood flow: **(A,B)** The PDA is seen with anterograde flow. **(C,D)** The PDA is seen with retrograde flow. Ao = aortic valve and ascending aorta, Ax = apex, D = spectral Doppler tracings of diastolic coronary blood flow, LA = left atrium, LV = left ventricle, RA = right atrium, S = spectral Doppler tracings of systolic coronary blood flow.

The methodology of visualization of the main trunks of the three coronary arteries has been described in detail previously [19]. In brief, the proximal left anterior descending coronary artery (LAD) could be seen from modified left parasternal short- and long-axis views, leaving the left main coronary artery (LM) and turning slightly toward the transducer. The course of the mid and distal LAD was imaged mostly from parasternal modified short- and long-axis views focusing on the anterior interventricular sulcus. From modified left parasternal short- and long-axis views, the left circumflex coronary artery (Cx) could be seen leaving the LM and further coursing in distal direction in the atrioventricular sulcus. From modified parasternal and subcostal short-axis, sagittal and 4-chamber views focusing on the tricuspid ring, the right coronary artery (RCA) could be traced leaving the aorta, passing the anterior surface of the tricuspid ring to the inferior margin of the right ventricle and further coursing on the medial and posterior tricuspid ring. The posterior descending coronary artery (PDA) could be imaged from modified apical 2-chamber views coursing toward the apex in the posterior interventricular sulcus (Figure 1A).

**Figure 2:**
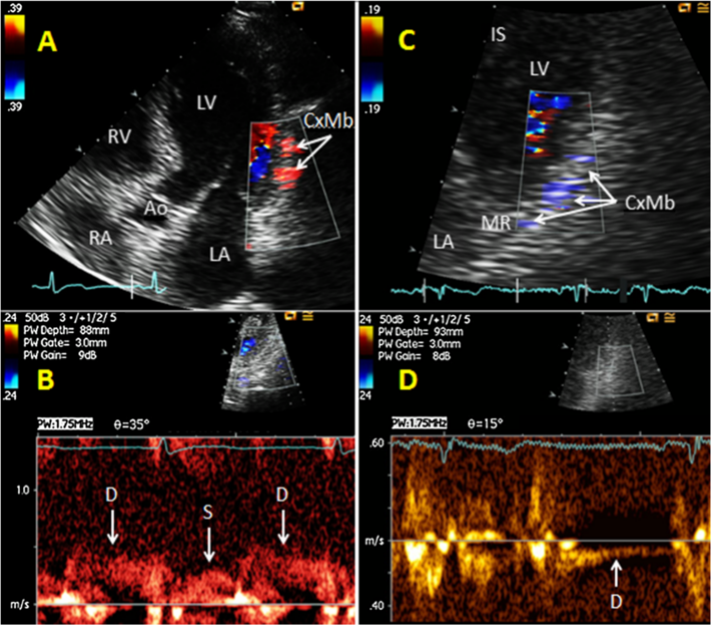
**Examples of anterograde and retrograde flow in marginal branches of the left circumflex coronary artery.** In modified apical four chamber views, a marginal branch of the left circumflex coronary artery (CxMb) is imaged by colour Doppler mapping with matching spectral Doppler tracings of blood flow: **(A,B)** The CxMb is seen with anterograde flow. **(C,D)** The CxMb is seen with retrograde flow. Ao = aortic valve and ascending aorta, D = spectral Doppler tracings of diastolic coronary blood flow, IS = interventricular septum, LA = left atrium, LV = left ventricle, MR = mitral ring, RA = right atrium, RV = right ventricle, S = spectral Doppler tracings of systolic coronary blood flow.

**Figure 3:**
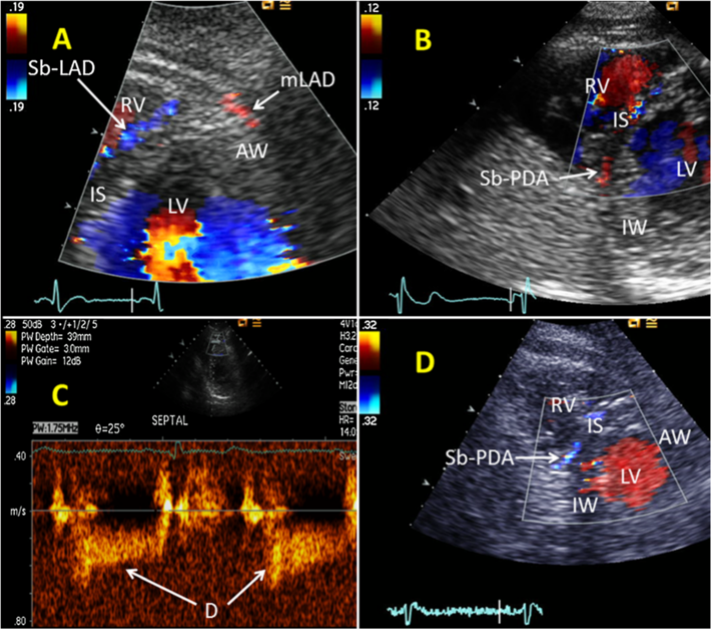
**Examples of normal anterograde and abnormal retrograde blood flow in septal perforating branches. (A)** In a modified parasternal short-axis view, a septal perforating branch from the left anterior descending coronary artery (Sb-LAD) is seen with anterograde blood flow. **(B)** In a modified parasternal short-axis view, a septal perforating branch from the posterior descending coronary artery (Sb-PDA) is seen with anterograde blood flow. **(C)** Spectral Doppler tracings of the anterograde blood flood in a Sb-LAD. **(D)** In a modified parasternal short-axis view, a Sb-PDA is seen with retrograde blood flow. AW = anterior wall of the left ventricle, D = spectral Doppler tracings of diastolic coronary blood flow, IS = interventricular septum, IW = inferior wall of the left ventricle, LV = left ventricle, mLAD = parts of the middle segment of the left anterior descending coronary artery, RV = right ventricle.

From modified apical four-chamber views focusing on different levels of the lateral wall of the left ventricle, marginal branches of Cx (CxMb) could be visualized running distally on the epicardial surface toward the transducer (Figure 2A). Using modified short-axis views, the interventricular septum was examined from base to apex for septal perforating branches from LAD (Sb-LAD) and PDA (Sb-PDA), with perforating branches located in the anterior half of septum defined as Sb-LAD and those in the posterior half of septum defined as Sb-PDA (Figures 3A and B).

**Figure 4:**
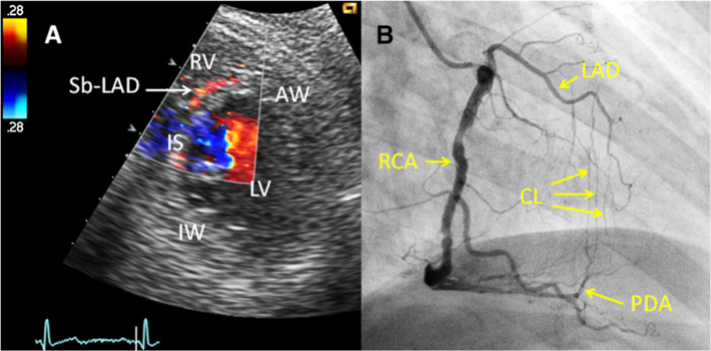
**Example of a transthoracic Doppler finding of retrograde collateral blood flow in a septal perforating branch, compared with coronary angiography. (A)** In a modified parasternal short-axis view, a septal perforating branch from the left anterior descending coronary artery (Sb-LAD) is seen with retrograde blood flow. **(B)** Angiogram of the right coronary artery (RCA) demonstrates collateral (CL) blood supply from the posterior descending coronary artery (PDA) through the septal perforating branches to the left anterior descending coronary artery (LAD). Angiogram of the left coronary artery demonstrated proximal LAD occlusion (not shown). AW = anterior wall of the left ventricle, IS = interventricular septum, IW = inferior wall of the left ventricle, LV = left ventricle, RV = right ventricle.

The epicardial coronary flow velocity waveform appears as a complex of a small wave in systole and a large trapezoid wave in diastole (Figures 1B and 2B). When colour Doppler recordings indicated reversed flow in a main coronary artery or CxMb thus making the functional diagnosis of upstream coronary occlusion [10,13-16] (Figures 1C and 2C), we distinguished the retrograde coronary flow from coronary venous flow by finding inverted coronary flow velocity waveform (Figures 1D and 2D). In contrast, the coronary venous flow appears as a prominent systolic wave. Retrograde flow in Sb-LAD and Sb-PDA was identified by colour Doppler (Figures 3D and 4A).

The flow velocity waveform of septal perforators is predominantly diastolic, with a small systolic wave either in the same or opposite direction, or lacking [20,21]. Measurements of systolic and mean diastolic flow velocities in the perforating vessels are significantly influenced by systolic cardiac motion and Doppler low velocity noise. Therefore, only peak diastolic flow velocities (pDV) were measured in these branches. When septal perforating branches demonstrated anterograde flow by colour Doppler (Figures 3A and B), their pDVs were measured using pulsed-wave Doppler with 1.75- to 3.5-Mhz frequency in a sample volume of 1.5 to 5 mm, with the sample volume positioned on the laminar colour flow Doppler signal (Figure 3C). We tried to find at least three consecutive cardiac cycles to average the flow velocities. Angle correction was used during velocity measurements to keep the angle between blood flow and Doppler beam as small as possible.

**Figure 5:**
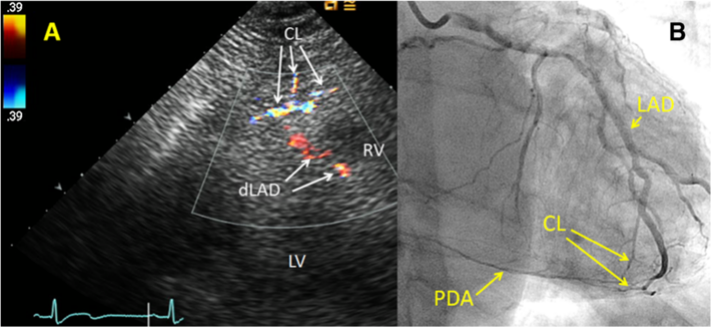
**Example of transthoracic Doppler findings of collateral blood flow in the apical region, compared with coronary angiography. (A)** In a modified parasternal long-axis view focusing on the apex and distal anterior interventricular sulcus, several epicardial and intramyocardial collaterals (CL) originates from the distal left anterior descending coronary artery (dLAD). **(B)** Angiogram of the left coronary artery demonstrates apically located epicardial and intramyocardial collateral (CL) blood supply from the dLAD to the posterior descending coronary artery (PDA), which shows retrograde flow. Angiogram of the right coronary artery demonstrated a proximal PDA occlusion (not shown). LAD = left anterior descending coronary artery, LV = left ventricle, RV = right ventricle.

Using standard and modified apical, parasternal, and subcostal views, the apex and free walls of the ventricles were scanned for epicardial and intramyocardial collaterals. In accordance with earlier studies [10,18], finding elongated epicardial or intramyocardial vessels with enhanced, typically mosaic-patterned flow was considered as collateral flow to an occluded coronary artery (Figure 5A).

### Coronary angiography

Coronary angiography was performed using standard techniques. All angiographic studies were digitally stored with later offline reviewing and analyses, blinded to the findings by TTE. Disagreements in interpretation were resolved by consensus between the two cardiologists responsible for the angiographic readings. All angiograms were classified according to left or right dominance. The severity of coronary stenoses in the LM and three major coronary arteries was determined by quantitative coronary angiography (QCA). The angiograms were analyzed using a 16-segment model of the coronary arteries [22]. Significant coronary disease was defined as diameter stenosis ≥ 50% in at least one main coronary artery. Each main coronary artery could have more than one stenosis, with the most severe lesion defining the degree of stenosis. Each coronary artery segment was categorized in one of the following four groups: (1) diameter stenosis 0% to 49%, (2) diameter stenosis 50% to 75% (borderline stenosis), (3) diameter stenosis 76% to 99% (high-grade stenosis), and (4) diameter stenosis 100% with focal absence of antegrade flow (occlusion). Coronary flow direction downstream to an occlusion was decided by following the coronary filling frame by frame. Collateral flow to occluded arteries was graded according to the Rentrop classification (grade 0 = no visible filling of any collateral channel, grade 1 = filling of side branches of the occluded artery, grade 2 = partial filling of the epicardial vessel, grade 3 = complete collateral filling of the epicardial vessel) [23].

### Reproducibility

To assess interobserver measurement variability, two experienced observers (J.V. and E.H.) examined data from 12 random cases in a blinded manner. Intraobserver variability was similarly tested by one experienced observer (J.V.) two weeks apart, blinded to previous results. For the reproducibility studies, minimum two separate septal perforator flow velocity waveform recordings were selected for measurements in each patient. Both interobserver and intraobserver measurement variability were expressed as the mean difference in percentage and the coefficient of variation of the differences between the measurements for each parameter.

### Statistical analysis

Continuous variables are presented as mean ± SD and categorical variables as fractions and percentages. Comparisons of mean values were performed using Student’s t tests for normally distributed parameters. Logistic regression analyses were used to explore relationships between the success rate for measurements of anterograde flow velocities in septal perforating branches and demographic and clinical variables, and to examine the different variables in predicting coronary occlusion, and confirmed by exact tests. Linear regression analyses were used to explore the relationships between anterograde flow velocities in septal perforating branches and baseline characteristics. Receiver operating characteristic (ROC) curve analysis was used to assess the optimal cutoff value of anterograde flow velocities in septal perforating vessels. Sensitivity, specificity, positive predictive value (PPV), and negative predictive value (NPV) to detect occlusion in the presence of at least one positive parameter indicating collateral flow were assessed using standard formulas. P values < 0.05 were considered statistically significant. All analyses were performed with SPSS for Windows version 20.0 (SPSS, Inc., Chicago, IL).

## Results

### Coronary angiography

There were 35 patients with and 73 patients without unstable coronary artery disease (Table 1). For stable patients, the mean time from echocardiographic examination to angiography was 24.7 ± 31.7 days. In the group with unstable coronary artery disease two patients had a postponed angiography due to unrelated comorbid conditions. The mean time from echocardiographic examination to angiography was 4.3 ± 3.4 days for the remaining 33 patients.

Eight patients had dominant Cx with PDA originating from the distal Cx, and in one patient, co-dominance was demonstrated by angiography. Table 2 lists findings on QCA for stenoses in the three main coronary arteries (segments 1–8, 11, 13, and 15 in the American Heart Association’s 16-segment model) [22] in stenosis groups 2 – 4. At angiography a total occlusion was demonstrated in 28 coronary artery segments. Three patients had two occluded arteries, while the rest had occlusion of a single artery. Twenty arteries showed retrograde flow at angiography and seven arteries showed anterograde flow downstream to the occlusion (Table 2), all with Rentrop grade ≥ 2 collateral circulation. One patient with a proximal LAD occlusion demonstrated ambiguous flow distal to the occlusion, with Rentrop grade 1 collateral circulation.

**Table 2:** Findings by angiography and QCA in the main coronary arteries

**Segment**	**Stenosis group 2**	**Stenosis group 3**	**Stenosis group 4**	**Blood flow direction in coronary trunk downstream to occlusion**	
	**(DS 50% – 75%)**	**(DS 76% – 99%)**	**(DS 100%; occlusion)**		
				**Retrograde**	**Anterograde**
LM	4	0	0		
pLAD	21	8	4	2	1
mLAD	15	3	2		2
dLAD	4	0	2		2
pCx	6	2	2		2
mCx	5	3	2	2	
dCx	3	2	0		
pRCA	14	7	6	6	
mdRCA	14	10	8	8	
PDA	4	1	2	2	

*dCx* distal left circumflex coronary artery, *dLAD* distal left anterior descending coronary artery, *DS* diameter stenosis, *LM* left main coronary artery, *mCx* middle left circumflex coronary artery, *mdRCA* mid-to-distal right coronary artery, *mLAD* middle left anterior descending coronary artery, *pCx* proximal left circumflex coronary artery, *PDA* posterior descending coronary artery, *pLAD* proximal left anterior descending coronary artery, *pRCA* proximal right coronary artery, *QCA* quantitative coronary angiography.

### TTE in demonstrating occluded main arteries

TTE showed retrograde flow in two LADs and six RCA/PDAs. Coronary angiography confirmed retrograde flow in all these arteries, except in two PDAs where anterograde flow was seen. This discrepancy was probably due to misinterpretation of LAD running around the apex. The sensitivity and specificity for identifying occlusion in arteries with angiographic downstream retrograde flow were 30% and 99%, respectively. Analyses showed no statistical differences in TTE detection of retrograde flow in a coronary artery when adjusted for baseline characteristics of the study cohort, with the exception of reduced feasibility in patients with acute coronary syndromes (ACS) (p = 0.02).

Retrograde flow was demonstrated by colour Doppler in Sb-LADs in five patients. Four of these patients had an upstream LAD occlusion, while one patient had an upstream high-grade LAD stenosis. Angiography showed, downstream to the four LAD occlusions, anterograde flow in three patients and retrograde flow in one patient. TTE showed retrograde flow in Sb-PDAs in 11 patients. RCA was occluded in proximal or mid segments in seven of these patients, while the remaining four patients showed upstream high-grade RCA stenoses. Retrograde flow in CxMb was found in one patient (Figures 2C and D), with angiography showing retrograde flow downstream to a mid Cx occlusion.

**Figure 6:**
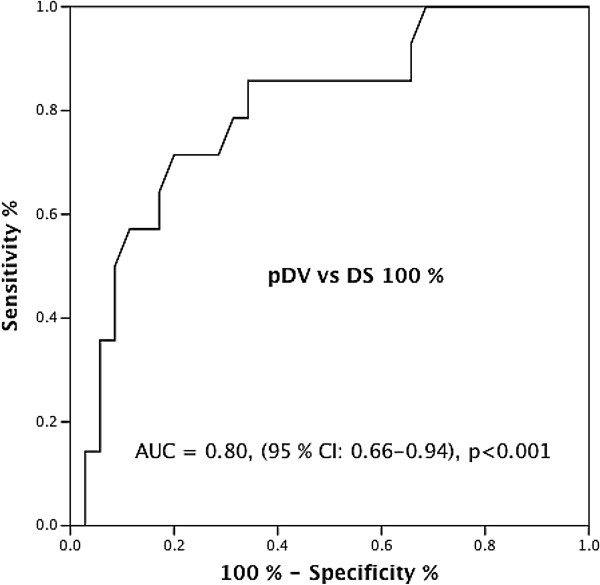
**ROC curve for anterograde peak diastolic flow velocities in septal perforators in diagnosing contralateral coronary occlusion.** Receiver operating characteristic (ROC) curve for anterograde peak diastolic flow velocities (pDV) in septal perforating branches in the diagnosis of occlusion in the contralateral, collateral-receiving main coronary artery, with optimal pDV ≥ 0.57 m/sec (sensitivity 79%, specificity 69%). AUC = area under the curve, CI = confidence interval, DS = diameter stenosis.

In patients with visible anterograde flow in septal perforating vessels, pDVs were measured in Sb-LADs in 47 of 49 patients (96%) and in Sb-PDAs in 2 of 3 patients. Coronary connections through the septum are mainly between the LAD and PDA, while collateral connections to the Cx territory mostly are epicardial [12]. Both borderline and high-grade stenoses in the ipsilateral and/or contralateral coronary artery might influence pDV. Four patient groups with pDV measurements were defined: group A = patients without significant coronary disease in the three main coronary arteries, group B = patients with borderline stenoses in the ipsilateral and/or contralateral coronary artery, group C = patients with high-grade stenoses in the ipsilateral and/or contralateral coronary artery, and group D = patients with occlusion in the contralateral coronary artery. Peak DVs measured in groups A - D are listed in Table 3. No statistically significant differences were found when comparing pDVs in groups A, B, and C, although there was a trend toward increasing pDV with increasing lesion severity in the contralateral artery. There was, however, statistically significant higher pDV in group D compared with pDV in group A (p < 0.001). Moreover, pDV was measured in Sb-LAD in two patients (pDV 1.63 m/sec and 0.68 m/sec, respectively) with occluded LAD without other significant coronary lesions. Both patients had intraseptal collateral bypass supplying blood through Sb-LADs originating upstream to the occlusion to Sb-LADs downstream to the occlusion, illustrating the diversity and complexity of coronary pathophysiology in the course of occluded coronary arteries. The ROC curve for pDV for the detection of coronary occlusion in the contralateral, collateral-receiving artery is shown in Figure 6. ROC curve analysis demonstrated that the optimal pDV cutoff value of 0.57 m/sec had a sensitivity of 79% and a specificity of 69% for detection of occlusion in the contralateral artery. Excluding Sb-PDA, the same pDV cutoff value for Sb-LAD had a sensitivity of 85% and a specificity of 70% for detection of occlusion in RCA. Two patients showed septal collateral flow with pDV ≥ 0.57 m/sec to a contralateral artery with upstream high-grade stenosis. The sensitivity and specificity of pDV above the cutoff value in the detection of diameter stenoses in the range 76% - 100% in the contralateral artery were 68% and 69%, respectively.

**Table 3:** Anterograde peak diastolic flow velocities (pDV) in septal perforating branches in groups A – D

**Groups**	**pDV (m/sec)**
Group A (n = 11)	0.37 ± 0.13
Group B (n = 8)	0.46 ± 0.28
Group C (n = 14)	0.50 ± 0.24
Group D (n = 14)	0.80 ± 0.31

**Group A** = patients without significant coronary disease in the three main coronary arteries.

**Group B** = patients with borderline stenoses (diameter stenosis, 50% - 75%) in the ipsilateral and/or contralateral coronary artery.

**Group C** = patients with high-grade stenoses (diameter stenosis, 76% - 99%) in the ipsilateral and/or contralateral coronary artery.

**Group D** = patients with occlusion in the contralateral coronary artery.

Excluding the septal collateral pathway, seven patients were identified by TTE having other intramyocardial or epicardial collaterals to occluded main coronary arteries. The most common finding was apically located epicardial and intramyocardial collaterals between the distal LAD and PDA (Figure 5A). Other findings were intramyocardial and epicardial free wall collaterals to myocardial territories originally supported by the LAD or Cx. These findings correctly identified the occluded coronary artery in all seven patients.

Findings of anterograde pDV ≥ 0.57 m/sec in septal branches, retrograde flow in a coronary artery or septal branch, or demonstration of other collaterals were all significantly related to angiographic occlusion (p < 0.001). The individual coronary occlusion could in our study be identified by at least one of the above mentioned TTE findings. Using these criteria, 25 of 28 coronary occlusions (89%) were correctly identified. Seven of eight LAD occlusions, two of four Cx occlusions, and all 16 RCA/PDA occlusions were correctly identified. Detecting at least one positive TTE parameter indicating occlusion had a sensitivity of 89%, specificity of 94%, PPV of 63%, and NPV of 99% for detection of coronary occlusion, as defined by angiography. Analyses showed no statistical differences in the degree of demonstrating collateral flow when adjusted for baseline characteristics of the study cohort, left or right dominance, Rentrop collateral flow, or clinical presentation. Seven arteries with high-grade stenosis demonstrated collateral flow to the artery downstream to the lesion (pDV ≥ 0.57 m/sec or retrograde flow in septal perforating branches). With the combined use of several TTE parameters indicating a coronary occlusion, the sensitivity, specificity, PPV, and NPV for detecting either an occlusion or a high-grade stenosis were 52%, 97%, 82%, and 88%, respectively.

### Observer variability

There was no significant difference in mean pDV value between the observers. The biases for pDV were 2.0% for intraobserver (p = 0.14) and 2.3% for interobserver measurements (p = 0.11). The intraobserver and interobserver coefficients of variation for pDV were 3.9% and 4.3%, respectively.

## Discussion

In this study of patients with suspected or definitive coronary artery disease, the feasibility and accuracy of diagnosing occlusions in the three main coronary arteries by use of transthoracic echocardiographic findings of collateral flow were examined and compared with findings at angiography. The main results were that (1) anterograde peak diastolic flow velocities (pDV) ≥ 0.57 m/sec in septal perforating vessels predicted contralateral coronary occlusion; (2) the combined use of several parameters indicating collateral flow identified 25 of 28 main coronary occlusions; (3) with TTE supplemental information may be obtained of the complex pathophysiology in patients with coronary occlusions.

We found significantly higher anterograde flow velocities in septal perforating branches in patients with contralateral coronary occlusion compared with corresponding velocities in patients without significant coronary disease (Table 3). In patients without coronary disease, other investigators have found pDV at 0.41 ± 0.13 m/sec [24], which is in agreement with our findings. Normally, a coronary vessel will dilate as an adaption to increased flow. It is anticipated that septal perforators with increased flow after achieving collateral function may not be capable of sufficient dilatation, possibly because of restraint from septal myocardium. This, in turn, leads to increased septal blood flow velocities to maintain the collateral function. A similar mechanism has been proposed to explain the increased flow velocities in septal perforators in hypertrophic cardiomyopathy [24]. In our study, the optimal pDV cutoff value ≥ 0.57 m/sec showed high sensitivity (79%) and moderate specificity (69%) for detection of coronary occlusion in the contralateral artery (Figure 6). Peak DV above the cutoff value also identified high-grade stenoses in the contralateral artery in some cases, and pDV ≥ 0.57 m/sec for detecting either coronary occlusion or high-grade stenosis in the contralateral artery had a sensitivity of 68%. To the best of our knowledge, our TTE study is the first to use a pDV cutoff value to identify coronary occlusions. However, this cutoff value needs to be tested in a separate study population.

Several investigators have shown that more coronary occlusions were detected when using two or even three parameters for identifying collateral flow compared to using only a single parameter [14-16]. By the combined use in our study of several parameters indicating collateral flow, we were able to correctly identify nearly all coronary occlusions. In accordance with findings in other studies [11,12], several high-grade stenoses in our study demonstrated collateral flow to jeopardized myocardium downstream to the lesion, illustrating the similar pathophysiology induced by high-grade stenoses and total occlusions. This explains that findings of collateral flow in our study showed moderate PPV (63%) for detection of coronary occlusion but high PPV (82%) for detecting either coronary occlusion or high-grade stenosis. In patients with ACS and an occluded culprit artery the time interval from the occlusion to the TTE investigation was short. This may have affected our results due to the time dependence of the collateral circulation to develop.

TTE correctly demonstrated retrograde flow in the coronary trunk in six of the twenty arteries with retrograde flow on coronary angiography, in two LADs and four PDAs. All six cases showed Rentrop class ≥ 2 collateral circulation. The sensitivity and specificity of diagnosing retrograde flow in main artery trunks were 30% and 99%, respectively, while other investigators have reported sensitivity and specificity of 70% to 100% and 96% to 100%, respectively [13-16]. The higher sensitivity in other studies may be because contrast agent was used to enhance Doppler signals and patients with ACS were not examined. In our study, a significant proportion of occluded coronary arteries showed collateral-dependent anterograde flow distal to the occlusion, a finding that has also been reported by other investigators [10]. Occluded coronary arteries with anterograde flow downstream to the occlusion will not be identified by only searching for retrograde flow in the main artery.

Retrograde blood supply through septal perforating branches was demonstrated in four of eight (50%) and seven of sixteen patients (44%) with occluded LADs and RCAs, respectively. In other TTE studies, reversed septal blood flow was demonstrated in 26% to 50% and 17% to 39% of patients with occluded LAD and RCA, respectively [14-16]. Collateral growth is also stimulated to support jeopardized myocardium downstream to severe stenoses or sub-total occlusions [11,12]. This probably explains our findings of five patients with high-grade stenosis in the LAD or RCA showing reversed flow in septal perforating branches.

In this study, finding epicardial and intramyocardial collaterals in the apex and free walls correctly identified coronary occlusions. Demonstration of free wall collaterals may be of special importance for diagnosing Cx occlusions because collateral flow to the Cx territory may be less using the intraseptal pathway.

### Clinical implication

The results from our TTE study indicate that findings of elevated anterograde flow velocities in septal perforators and the combined use of several parameters indicating collateral flow are valuable methods for identifying coronary occlusions, with the possibility of identifying most of these occlusions using several parameters. Our study also demonstrates the heterogeneity and complexity of coronary flow physiology that may occur in patients with coronary occlusions. In our experience, the TTE examination for coronary occlusions takes 15 to 20 minutes.

### Study limitations

There are several limitations in our study. Because of longer distance to the echocardiographic probe, the Cx, CxMbs, RCA/PDA, and septal branches from PDA were more difficult to visualize than the LAD and its septal branches. The use of ultrasound contrast agent might have improved the feasibility of demonstrating collateral and retrograde flow [14,15]. Because of limited clinical experience in patients with ACS, we chose not to use ultrasound contrast when planning this study. The response in pDV is probably not binary (normal or abnormal) or absolute for the pDV cutoff value of ≥ 0.57 m/sec, implying that pDV measurements slightly below or above the cutoff value might give uncertain estimates of coronary occlusion. Future studies with larger patient cohorts may possibly refine our proposed pDV cutoff value. Finally, we cannot exclude selection bias, because our study cohort included only patients planned for coronary angiography and excluded those with previous coronary artery bypass surgery, presumed insufficient acoustic windows, significant valvular disease, or atrial fibrillation.

## Conclusions

We found that an anterograde peak diastolic flow velocity of ≥ 0.57 m/sec in septal perforating branches showed high ability to identify occlusion in the contralateral main coronary artery. With the combined use of several parameters indicating collateral flow, we were able to identify the majority of coronary occlusions in our patient cohort. Moreover, investigating collateral flow can give further insight into the complex coronary flow physiology that may result from coronary artery occlusions.

## Competing interests

The authors declare that they have no competing interests.

## Authors’ contribution

JV conceived the study, carried out the ultrasound examinations and drafted the manuscript. EH carried out the ultrasound examinations, helped to perform the statistical analyses and draft the manuscript. RW carried out the angiography readings and helped to draft the manuscript. KH carried out the angiography readings. TH participated in the design of the study and helped to perform the statistical analyses and draft the manuscript. All the authors read and approved the final manuscript.

## References

[B1] Christofferson RD Lehmann KG Martin GV Every N Caldwell JH Kapadia SR Effect of chronic total coronary occlusion on treatment strategy Am J Cardiol 2005 95 1088 1091 10.1016/j.amjcard.2004.12.065 15842978

[B2] Kahn JK Angiographic suitability for catheter revascularization of total coronary occlusions in patients from a community hospital setting Am Heart J 1993 126 561 564 10.1016/0002-8703(93)90404-W 8362709

[B3] Puma PA Sketch MH Jr Tcheng JE Gardner LH Nelson CL Phillips HR Stack RS Califf RM The natural history of single-vessel chronic coronary occlusion: a 25-year experience Am Heart J 1997 133 393 399 10.1016/S0002-8703(97)70179-0 9124159

[B4] Trappe HJ Lichtlen PR Klein H Wenzlaff P Hartwig CA Natural history of single vessel disease. Risk of sudden coronary death in relation to coronary anatomy and arrhythmia profile Eur Heart J 1989 10 512 524 10.1093/oxfordjournals.eurheartj.a059521 2759112

[B5] van der Schaaf RJ Vis MM Sjauw KD Koch KT Baan J Jr Tijssen JGP de Winter RJ Piek JJ Henriques JPS Impact of multivessel coronary disease on long-term mortality in patients with ST-elevation myocardial infarction is due to the presence of a chronic total occlusion Am J Cardiol 2006 98 1165 1169 10.1016/j.amjcard.2006.06.010 17056319

[B6] Meier P Hemingway H Lansky AJ Knapp G Pitt B Seiler C The impact of the coronary collateral circulation on mortality: a meta-analysis Eur Heart J 2011 33 614 621 21969521 10.1093/eurheartj/ehr308

[B7] Meier P Seiler C The coronary collateral circulation - clinical relevances and therapeutic options Heart 2013 99 897 898 10.1136/heartjnl-2012-303426 23263705

[B8] Piek JJ van Liebergen RAM Koch KT Peters RJG David GK Clinical, angiographic and hemodynamic predictors of recruitable collateral flow assessed during balloon angioplasty coronary occlusion J Am Coll Cardiol 1997 29 275 282 10.1016/S0735-1097(96)00499-8 9014978

[B9] Mills JD Fischer D Villanueva FS Coronary collateral development during chronic ischemia: serial assessment using harmonic myocardial contrast echocardiography J Am Coll Cardiol 2000 36 618 624 10.1016/S0735-1097(00)00739-7 10933379

[B10] Pizzuto F Voci P Puddu PE Chiricolo G Borzi M Romeo F Functional assessment of the collateral-dependent circulation in chronic total coronary occlusion using transthoracic Doppler ultrasound and venous adenosine infusion Am J Cardiol 2006 98 197 203 10.1016/j.amjcard.2006.01.075 16828592

[B11] Rigo P Becker LC Griffith LSC Alderson PO Bailey IK Pitt B Burow RD Wagner HN Jr Influence of coronary collateral vessels on the results of thallium-201 myocardial stress imaging Am J Cardiol 1979 44 452 458 10.1016/0002-9149(79)90396-5 474425

[B12] Levin DC Pathways and functional significance of the coronary collateral circulation Circulation 1974 50 831 837 10.1161/01.CIR.50.4.831 4425386

[B13] Watanabe N Akasaka T Yamaura Y Akiyama M Koyama Y Kamiyama N Neishi Y Kaji S Saito Y Yoshida K Noninvasive detection of total occlusion of the left anterior descending coronary artery with transthoracic Doppler echocardiography J Am Coll Cardiol 2001 38 1328 1332 10.1016/S0735-1097(01)01556-X 11691503

[B14] Hirata K Watanabe H Hozumi T Tokai K Otsuka R Fujimoto K Shimada K Muro T Yoshiyama M Yoshikawa J Simple detection of occluded coronary artery using retrograde flow in septal branch and left anterior descending coronary artery by transthoracic Doppler echocardiography at rest J Am Soc Echocardiogr 2004 17 108 113 10.1016/j.echo.2003.09.019 14752483

[B15] Otsuka R Watanabe H Hirata K Tokai K Muro T Hozumi T Yoshiyama M Yoshikawa J A novel technique to detect total occlusion in the right coronary artery using retrograde flow by transthoracic Doppler echocardiography J Am Soc Echocardiogr 2005 18 704 709 10.1016/j.echo.2004.11.018 16003266

[B16] Boshchenko AA Vrublevsky AV Karpov RS Transthoracic echocardiography in the detection of chronic total coronary artery occlusion Eur J Echocardiogr 2009 10 62 68 10.1093/ejechocard/jen159 18490275

[B17] Saraste M Vesalainen RK Ylitalo A Saraste A Koskenvuo JW Toikka JO Vaittinen M-A Hartiala JJ Airaksinen KEJ Transthoracic Doppler echocardiography as a non-invasive tool to assess coronary artery stenoses - a comparison with quantitative coronary angiography J Am Soc Echocardiogr 2005 18 679 685 10.1016/j.echo.2004.09.016 15947773

[B18] Anjaneyulu A Johann C Raghava RP Kumar DN Sridevi C Soma RB Krishnam RP Rajagopala RA Coronary collaterals by transthoracic echocardiography in coronary artery disease J Am Soc Echocardiogr 2004 17 466 469 10.1016/j.echo.2003.12.019 15122189

[B19] Vegsundvåg J Holte E Wiseth R Hegbom K Hole T Transthoracic echocardiography for imaging of the different coronary artery segments: a feasibility study Cardiovasc Ultrasound 2009 7 8 10.1186/1476-7120-7-8 20028530 PMC2806270

[B20] Chilian WM Marcus ML Effects of coronary and extravascular pressure on intramyocardial and epicardial blood velocity Am J Physiol 1984 248 H170 H178 3970221 10.1152/ajpheart.1985.248.2.H170

[B21] Youn HJ Redberg RF Schiller NB Foster E Demonstration of penetrating intramyocardial coronary arteries with high-frequency transthoracic echocardiography and Doppler in human subjects J Am Soc Echocardiogr 1999 12 55 63 10.1016/S0894-7317(99)70173-2 9882779

[B22] Austen WG Edwards JE Frye RL Gensini GG Gott VL Griffith LS McGoon DC Murphy ML Roe BB A reporting system on patients evaluated for coronary artery disease. Report of the Ad Hoc Committee for Grading of Coronary Artery Disease, Council on Cardiovascular Surgery, American Heart Association Circulation 1975 51 suppl 5 40 1116248 10.1161/01.cir.51.4.5

[B23] Rentrop KP Cohen M Blanke H Phillips RA Changes in collateral channel filling immediately after controlled coronary artery occlusion by an angioplasty balloon in human subjects J Am Coll Cardiol 1985 5 587 592 10.1016/S0735-1097(85)80380-6 3156171

[B24] Sherrrid MV Mahenthiran J Casteneda V Fincke R Gasser M Barac I Thayaparan R Chaudhry FA Comparison of diastolic septal perforator flow velocities in hypertrophic cardiomyopathy versus hypertensive left ventricular hypertrophy Am J Cardiol 2006 97 106 112 10.1016/j.amjcard.2005.07.128 16377293

